# Apolipoprotein E4 allele is genetically associated with risk of the short- and medium-term postoperative cognitive dysfunction: A meta-analysis and trial sequential analysis

**DOI:** 10.1371/journal.pone.0282214

**Published:** 2023-02-24

**Authors:** Wei-Jen Hsiao, Chien-Yu Chen, Yi-No Kang, Chaur-Jong Hu, Che-Hong Chen, Pei-Lin Lin, Yu-Cih Lin

**Affiliations:** 1 School of Medicine, College of Medicine, Taipei Medical University, Taipei, Taiwan; 2 Department of Anesthesia, Taipei Medical University Hospital, Taipei, Taiwan; 3 Department of Anesthesia, School of Medicine, College of Medicine, Taipei Medical University, Taipei, Taiwan; 4 Graduate Institute of Humanities in Medicine, College of Humanities and Social Sciences, Taipei Medical University, Taipei, Taiwan; 5 Evidence-Based Medicine Center, Wan Fang Hospital, Taipei Medical University, Taipei City, Taiwan; 6 Cochrane Taiwan, Taipei Medical University, Taipei City, Taiwan; 7 Institute of Health Policy and Management, College of Public Health, National Taiwan University, Taipei City, Taiwan; 8 Department of Neurology, Shuang Ho Hospital, Taipei Medical University, New Taipei City, Taiwan; 9 Department of Chemical and Systems Biology, School of Medicine, Stanford University, Stanford, California, United States of America; 10 Department of Anesthesia, National Taiwan University Hospital, Taipei, Taiwan; 11 School of Nursing, College of Nursing, Taipei Medical University, Taipei, Taiwan; 12 Post-Baccalaureate Program in Nursing, College of Nursing, Taipei Medical University, Taipei City, Taiwan; James Cook University, AUSTRALIA

## Abstract

The aim of systematic review and meta-analysis was to investigate whether APOE4 was associated with postoperative neurologic dysfunction occurrence in short- or medium-term among surgical patients and to study the potential genetic association among these two entities. We searched electronic databases for reserch studies to evaluate the association of APOE4 with postoperative delirium (POD) or short- and medium term postoperative cognitive dysfunction (POCD). Twenty-two trials (16 prospective and six retrospective) with 6734 patients were included. APOE4 alleles was shown significantly associated with POCD within 1 week (odds ratio, OR, 1.89, 95% confidence interval, CI, 1.36 to 2.6278, p < 0.01) in the random-effects model. A significant association was also noted between APOE4 and POCD in medium-term, 1–3 months, after surgery (OR: 1.67, 95% CI: 1.003–2.839, p = 0.049). However, APOE4 was not significantly associated with POCD 1 year after surgery (OR: 0.98, 95% CI: 0.57–1.70, p = 0.9449) and POD (OR: 1.28, 95% CI: 0.85–1.91, p = 0.23). In conclusion, APOE4 alleles was genetically associated with short- and medium-term postoperative neurological dysfunction and future screening or preventive strategies derived is highly potential to improve outcomes.

## Introduction

Postoperative delirium (POD) and postoperative cognitive dysfunction (POCD) are two major neurological morbidities after patients receiving surgery and anesthesia which were significantly distressing to patients, their families, and caregivers. POD is defined as the clinical syndrome of transient fluctuating disturbance in attention, mental status and consciousness, occurred immediate postoperatively after anesthesia and surgery up to during hospitalization and its incidence ranging from 3–53% [1–6]. Unlike POD being diagnosed with clinical symptoms, POCD is defined by neuropsychological tests including different domains of impaired cognition, such as verbal memory, visual memory, language comprehension, visuospatial abstraction, attention, and concentration, incidence ranging 9.9–45% in the short (7 days) and medium (1–3 months) term even till 7.5 years after anesthesia and surgery [1, 2, 6]. Either POD or POCD, increases the social burden and the mortality of patients after receiving surgery and anesthesia [3, 7–9].

The predisposing risk factors for POD or POCD have been studied in recent decade. Most previous studies focused on the patient’s pre-existing morbidities (eg. neurological disorders) [10], surgical procedures (eg. cardiac surgery using extracorporeal cardiopulmonary bypass) [11], anesthetic techniques [12, 13] or various anesthetics, either totally intravenous (TIVA) or inhalational [14, 15], has also been recently hypothesized to be associated with POCD or POD. Recently, patients’ biological predisposing risk factors such as genetic factor for POCD or POD cause researchers’ interest and this might provide new domain developing screening or preventive strategies [16].

Neuro-inflammation and deposition of amyloid-β contributing the mechanisms for Alzheimer’s disease (AD), the most common disease of cognitive impairment in human, was also be involved in POCD [17]. The apolipoprotein E (APOE) gene lies in chromosome 19 containing three polymorphic alleles, namely ɛ2, ɛ3, and ɛ4 [18] and the ɛ4 allele (APOE4) is involved in amyloid plaque deposition playing a major role for AD and other neurological disorders [19]. The association of APOE4 and POD has also been noted in previous study [20]. The frequencies of APOE4 allele expression with racial specificity in African American, Caucasian, Hispanic, and Japanese individuals were approximately 26%, 24%, 15%, and 12%, respectively [21]. Previous meta-analysis showed a significant association between APOE4 and POCD a week after surgery but exhibited non-significant 1 month to 1 year after surgery [22].

However, they included only few studies and being interfered by one study with predominant patient number included and there are additional studies being published afterwards [23–25]. Previous meta-analyses have reported no association between APOE4 and delirium [26, 27]. However, no meta-analysis has examined the relationship between APOE4 and POD. Although the first relevant cohort study reported a significant association of APOE4 with delirium in patients undergoing non-cardiac surgery [28], further studies in patients receiving vascular, gastrointestinal or orthopedic surgeries showed controversy [29, 30].

Thus, the aim of systematic review and meta-analysis was to investigate whether APOE4 was associated with POD or POCD occurrence in short- or medium-term among surgical patients and searching with trial sequential analysis in order to study the potential genetic association among these two entities.

## Methods

### Registration and protocol

This study was conducted in accordance with Preferred Reporting Items for Systematic Reviews and Meta-Analyses guidelines [31, 32] and the meta-analysis of observational studies in epidemiology checklist [32, 33]. The study protocol was registered in the International Prospective Register of Systematic Reviews (PROSPERO) on June 20, 2021 (registration number: CRD42021256262).

### Eligibility criteria

We included studies investigating patients receiving surgery under general or regional anesthesia and those harboring the APOE4 allele and evaluated with at least one cognitive function test or postoperative neurological outcome. Exclusion criteria were as follows: (i) study without comparison of APOE4 and non-APOE4; (ii) POCD and POD were not confirmed by accepted criteria such as Mini-Mental State Examination, International Study of Postoperative Cognitive Dysfunction, Delirium Rating Scale, and other commonly used tool or methods in clinical practice; (iii) studies without data on outcomes of interests for both APOE4 and non-APOE4 groups; (iv) study with non-comparative design in terms of not case-control design; (v) studies of pediatric patients or those undergoing brain surgery; and (vi) studies of case reports or series without one-year follow-up.

### Search strategy and study selection

Two independent reviewers (YC Lin and WJ Hsiao) systematically searched for relevant studies published until July 2, 2021, in PubMed, Embase, Web of Science, Cochrane Library, CINAHL Plus, and PsycInfo by using the following medical subject headings: “postoperative cognitive decline” OR “postoperative cognitive complications” OR “postoperative cognitive dysfunction” OR “postoperative delirium” OR “acute delirium” OR “delirious syndrome” OR “confusion” OR “hallucinations” OR “inattentiveness” OR “disorientation” OR “illusions” OR “agitation” and with “apoe” OR “apoe4” OR “apo e” OR “apo e4” OR “apolipoprotein e” OR “apolipoprotein e4”(see S1 File, which illustrates the search strategy for all electronic databases).

The titles, abstracts, and full texts of studies were screened to evaluate whether they met the inclusion criteria. First, two reviewers (YC Lin and WJ Hsiao) screened titles and abstracts. Subsequently, when studies were likely to be related to our topic, the full text of the articles was reviewed. Finally, the third reviewer (PL Lin) resolved disagreements when the two reviewers had differences of opinion. Furthermore, when multiple studies shared the same patient source, we initially selected the study reporting the exact number of patients with POD or POCD with or without APOE4 expression or the study with a larger sample size. In addition, we manually searched the reference lists of included articles to find relevant studies by using Endnote (version X7, Clarivate Analytics, Philadelphia, PA).

### Data extraction

Two researchers (YC Lin and WJ Hsiao) retrieved the following data from selected studies by using a predetermined data extraction form: publication details; study design; number of participants; participants’ characteristics (age, sex, country, ethnicity, and comorbidities); types of surgery and anesthesia; genotyping methods and information; criteria and tools used for assessing POD or POCD; length of follow-up; and outcomes of interest. The two main outcomes of interest were 1) the incidence of POCD diagnosed using neuropsychological tests within 1 year after surgery, and 2) the incidence of POD diagnosed using objective criteria based on particular symptoms (e.g., disorientation, confusion, loss of attention, and hyperactive or hypoactive behaviors) within 1 week after surgery or before discharge.

### Quality assessment

The quality of included studies was examined using the eight-item Newcastle–Ottawa Scale (NOS) for cohort studies [33]. This tool assesses the following eight potential causes of bias in three domains: representativeness of exposure, selection of the non-exposed, ascertainment of exposure, absence of the outcome in the beginning, comparability of cohorts, assessment of outcomes, follow-up length, and adequacy of follow-up. Studies with an NOS score of ≥ 7 were considered to have high quality, whereas those with an NOS score of ≤ 3 were considered to have poor quality. Two reviewers (YC Lin and WJ Hsiao) examined the study quality, and the final decision was made by the third reviewer (CY Chen) in cases of disagreement between the two reviewers.

### Statistical analysis

Main analyses were carried out using R (version 4.1.0) via RStudio (RStudio Team, 2021, version 1.4.1717) [34]. All data were pooled based on random-effects model, which mainly based on the method proposed by Paule and Mandel (PM method) rather than DerSimonian-Laird due no requirement of distributional assumptions with less biased estimation than other alternatives [35]. The odds ratio (OR) was calculated to examine the effects of APOE4 on POD or POCD. Effect estimates were reported with summary OR and its 95% confidence intervals (95% CIs) and presented as forest plots. Owing to unavoidable clinical heterogeneity across observational studies, this study pooled data in random-effects model. Beside of clinical heterogeneity, statistical heterogeneity was detected using*I*^2^ statistics that defined as low, moderate, and high corresponding to the cut-off values of 25%, 50%, and 75%, respectively. Small-study effect was examined by evaluating the symmetry of the funnel plot and performing Egger’s test for each outcome. To detect source of ‘between-study heterogeneity’, sensitivity analysis study design, surgical type, and participants. Pooled analysis with forest plot were carried out by function “*metabin*” and “*forest*” in package “*meta*” version 6.0.0. With regard to small-study effects and funnel plot were illustrated using function “regtest” and “funnel” in package “metafor” version 3.8.1.

To reduce the risk of random errors and increase the robustness of the meta-analyses, we applied trial sequential analysis (TSA) for combining information size estimation (cumulated sample size of included trials) with an adjusted threshold for statistical significance in the cumulative meta-analysis which called trial sequential monitoring boundaries [36]. TSA reduces the risk of random error, increases the robustness of the meta-analyses, and determines whether the current sample size is adequate enough to draw a conclusion. We calculated the alpha-spending required information size (RIS) based on prior information from random-effects model due to heterogeneity across studies with α = 0.05 (two sided) and β = 0.10 (power = 90%) which would be more robust. More details about TSA are available elsewhere [36].

## Results

### Study selection and patient characteristics

**Fig 1:**
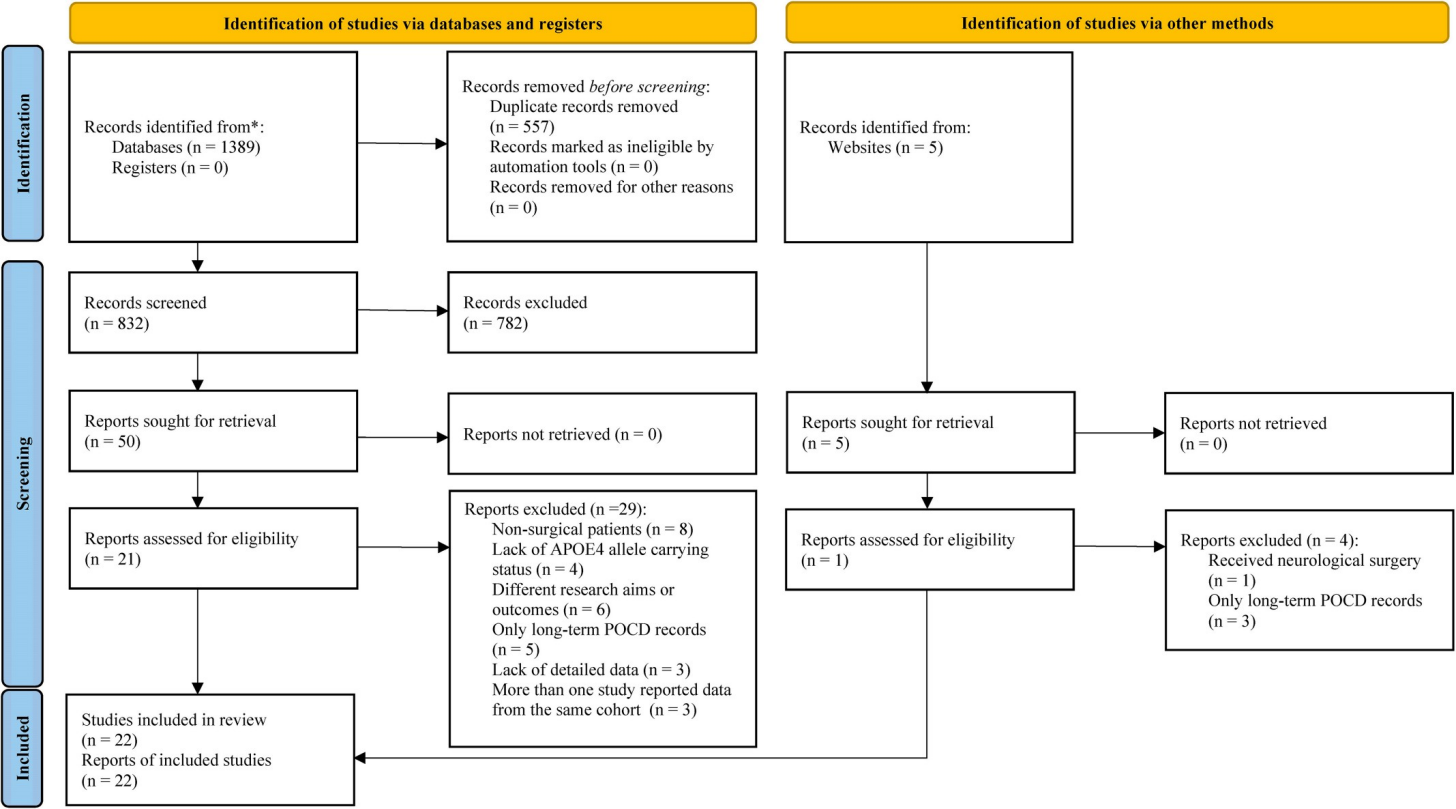
Flow diagram showing study selection process.

Fig 1 shows the flow diagram of the systematic review process used to identify relevant studies. After removing duplicate studies, we screened and evaluated 837 studies. Although 55 full-text studies initially met the inclusion criteria, 33 of them were excluded (see S1 Table which illustrates details of 33 full-text studies excluded). Final twenty-two studies with 6734 patients (2235 APOE4 carriers and 4499 non-carriers) were included in English from 2001 to 2018. Among them, 16 studies [23, 25, 28–30, 37–47] were prospective and six [24, 48–52] were retrospective in nature. The sample sizes of the included studies ranged from 33 to 2000 [40, 44], and the mean age of participants ranged from 62.18 to 74.4 years. Furthermore, 13 studies included patients who underwent cardiac or vascular surgery [25, 38, 39, 41–43, 45–47, 49–52]. Two studies [28, 51] did not exclude patients with pre-existing cognitive impairment, three studies [43, 46, 49] excluded only those with psychiatric illness, and three studies [41, 50, 52] did not mention whether patients were excluded according to cognitive status. Seven studies were conducted in the United States [23–25, 28, 29, 43, 49], four in the United Kingdom [30, 44, 50], three in Germany [41, 46, 47], two in Canada [39, 52], and one each in Portugal [37], Denmark [48], Turkey [38], China [40], Brazil [42], and Australia [51]. The postoperative follow-up length ranged from 1 day to 1 year. In terms of study quality, more than a third of the included studies (9 studies) had high quality (NOS ≥ 7), and no study had poor quality. Tables 1 and 2 summarize the detailed characteristics and patient demographics of the 22 studies, respectively.

**Table 1:** Characteristics of included studies.

Study	Design	Baseline age (median, IQR or mean ± SD)		Male (%)	Type of surgery	Type of anesthesia	Criteria for POD or POCD	Genotype Method	APOE4		Length of follow-up
									Carrier	Non-carrier	
**Abelha, 2012 [37]**	Prospective cohort	64 (51–74)		92 (53)	Noncardiac and nonneurological surgery	General or regional anaesthesia, or combined	ICDSC (POD)	PCR^a^	24	149	24 h and then every 8 h; mean days (IQR): 20 days (15–40)
**Abildstrom, 2004 [48]**	Retrospective cohort	65 (Not clear)		343 (35.1)	Noncardiac and nonneurological surgery	General or regional anaesthesia	ISPOCD (POCD)	PCR^b^	247	648	1 week and 3 months
Askar, 2005 [38]	Prospective cohort	apoE4 (+):	62.18 ± 8.45	63 (80.8)	Cardiovascular surgery	General anaesthesia	Cognistat (POCD)	Real-time^C^PCR, FRET	17	56	Discharge and 3 months
		apoE4 (-):	62.34 ± 9.64								
Bryson, 2011 [39]	Prospective cohort	apoE4 (+):	69.7 ± 6.2	64 (73.6)	Elective open abdominal aortic repair	Combined epidural and general anaesthesia	CAM (POD), Battery of nine neuropsychometric tests (POCD)	Blood sample^c^	20	63	Discharge and 3 months
		apoE4 (-):	71.6 ± 5.8								
**Cai, 2012 [40]**	Prospective study	70.1 ± 4.6		1140 (57)	Esophagectomy, gastrectomy, nephrectomy or fracture reduction	General anaesthesia	MMSE (POCD)	PCR^d^	574	1426	1 day
**Cunningham, 2017 [30]**	Prospective cohort	74.4 ± 5.8	136 (43.2)	Elective hip or knee arthroplasty	Spinal anaesthesia	CAM (POD), MMSE (severity of cognitive impairment)	TaqMan SNP Genotyping assays^e^	78	233	3 days
Deiner, 2015 [24]	Retrospective cohort	POCD (+):	73.7 ± 3.5	40 (51.9)	Noncardiac thoracic surgery	General anaesthesia with total intravenous anaesthesia	Cognitive tests of 5 domains (POCD)	Not clear	14	63	3 months
		POCD (-):	74.0 ± 5.4								
Guenther, 2013 [41]	Prospective cohort	POD (+):	73.3	143 (66.5)	Cardiovascular surgery	General anaesthesia	CAM-ICU (POD)	PCR^f^	63	128	1 week
			(71.2–75.4)								
		POD (-):	68.5								
			(67.0–70.0)								
**Heyer, 2005 [49]**	Retrospective cohort	69.1 ± 8.5		46 (61)	Carotid endarterectomy	General anaesthesia	5 standard neuropsychological tests (POCD)	PCR^d^	12	63	1 month
**Klinger, 2018 [25]**	Prospective cohort	69 ± 6		29 (76.3)	Cardiovascular surgery	General anaesthesia	Cognitive tests of 5 domains (POCD)	PCR^g^	8	29	6 weeks
Lelis, 2006 [42]	Prospective cohort	apoE4 (+):	65.5 ± 10.8	68 (78.2)	Cardiovascular surgery	General anaesthesia	MMSE (POCD)	PCR^f^	19	68	1 and 6 days
		apoE4 (-):	64.6 ± 9.1								
**Leung, 2007 [28]**	Prospective cohort	72.5 ± 5.9		98 (51.5)	Major non-cardiovascular surgery	General, regional anaesthesia, or combined	CAM (POD)	PCR^f^	46	144	2 days
**McDonagh, 2010 [43]**	Prospective cohort	68.1 ± 8.3		184 (50.7)	Noncardiovascular surgery	General or regional anaesthesia	Cognitive tests of 5 domains (POCD)	PCR^h^	76	274	6 weeks and 1 year
**Rentowl, 2004 [44]**	Prospective cohort	71 ± 6		25 (47)	Noncardiac thoracic surgery	General anaesthesia	ISPOCD (POCD)	Blood samples^c^	8	25	1 week and 3 months
**Robson, 2002 [50]**	Retrospective cohort	Not clear		Not clear	Cardiovascular surgery	General anaesthesia	6 cognitive tests (POCD)	Blood samples^c^	34	52	3 months
**Shoair, 2015 [23]**	Prospective cohort	71 ± 5.4		23 (33.3)	Elective knee or hip joint replacement, simple posterior cervical or lumbar spine surgery	General, regional anaesthesia, or combined	5 cognitive tests (POCD)	Blood samples^c^	14	54	3 months
**Silbert, 2008 [51]**	Retrospective cohort	68.4 ± 7.8		213 (75.5)	Cardiovascular surgery	General anaesthesia	8 cognitive tests (POCD)	PCR^i^	83	199	3 months and 1 year
Steed, 2001 [45]	Prospective study	apoE4 (+):	64.1 ± 6.1	96 (86.5)	Cardiovascular surgery	General anaesthesia	9 cognitive tests (POCD)	PCR^j^	30	81	6 weeks
		apoE4 (-):	64.7 ± 8.1								
Stewart, 2013 [52]	Retrospective study	POCD (+):	68 ± 5	139 (89.7)	Cardiovascular surgery	General anaesthesia	12 cognitive tests (POCD)	Sequenom Mass Array platform, RFLP^k^	33	122	1 year
		POCD (-):	67 ± 7								
**Tagarakis, 2007 [46]**	Prospective cohort	69.5 ± 7.7		99 (72.2)	Cardiovascular surgery	General anaesthesia	MMSE (POCD), WMS-R (POCD),BPRS (POCD), DRS (POD)	Rapid-cycle PCR, FRET with LightCycler	33	104	1 month (on any postop day)
**Vasunilashorn, 2015 [29]**	Prospective cohort	76.6 ± 5.2		233 (41.8)	Non-cardiovascular surgery	Not clear	CAM (POD) or a validated chart-review method (POD)	PCR^l^	108	449	Until discharge, more than 3 days
**Zoll, 2009 [47]**	Prospective cohort	67.9 ± 12.7		75 (76.5)	Cardiovascular surgery	Not clear	MMST (POCD), clock drawing (POCD), CAM (POD), IQCODE (POCD)	DNA hybridization^m^	29	69	Before discharge, 3 and 6 months and 1 year

^a^ PCR by sequencing was performed using the ABI Prism Dye Terminator Cycle Sequencing kit and an Applied Biosystems 3130xl Genetic Analyzer.

^b^ PCR was performed using combined HaeII and AflIII enzyme digestion, followed by separation on a 3% Metaphor agarose

^c^ Genotyping method was not clear.

^d^,PCR was performed through restriction fragment length polymorphism analysis by using DNA extracted from buffy coats of whole blood samples.

^e^ TaqMan SNP Genotyping Assay includes two differentially labeled, allele-specific TaqMan MGB probes and a PCR primer pair that uniquely amplify and provide unmatched specificity for the allele of interest.

^f^ Restriction isotyping of human apolipoprotein E by gene amplification and cleavage with HhaI.

^g^ PCR was performed using restriction enzyme isotyping.

^h^ PCR was used to amplify a short polymorphic region residing within the coding sequences of the human APOE gene.

^i^ Genomic DNA was extracted using the Blood Mini-Kit (Qiagen, Hilden, Germany).

^j^ PCR was performed for the rapid determination of the APOE genotype by using a heteroduplex generator.

^k^ Apolipoprotein E alleles were determined by the combinations of two SNPs (NCBI-dbSNP, rs7412 and rs429358) and genotyping for these two APOE polymorphisms was performed through restriction fragment length polymorphism analysis.

^l^ PCR was performed through simplified universal genomic DNA extraction.

^m^ The APOE4 genotype was determined using DNA hybridization (Innogenetics, Belgium).

+, Positive; −, Negative; apoE4, apolipoprotein E4; BPRS, Brief Psychiatric Rating Scale; CAM, Confusion Assessment Method; CAM-ICU, Confusion Assessment method for the Intensive Care Unit; DRS, Delirium Rating Scale; FRET, fluorescence resonance energy transfer; IQR, interquartile range; ICDSC, Delirium Screening Checklist; ISPOCD, International Study of Postoperative Cognitive Dysfunction; IQCODE, Informant Questionnaire on cognitive decline; MMSE, Mini-Mental State Examination [53]; MMST, Mini-Mental-Status Test; PCR, polymerase chain reaction; POCD, postoperative cognitive dysfunction; POD, postoperative delirium; RFLP, restriction fragment length polymorphism analysis; SD, standard deviation; SNP, single-nucleotide polymorphism genotyping assay; WMS-R, Wechsler Memory Scale Revised

**Table 2:** Critical appraisal of included studies.

Study	Country	Patients with cognitive impairment excluded	Selection				Comparability	Outcome			Quality Score
			Representativeness	Selection of the nonexposed	Ascertainment of exposure	Outcome of interest not present at start of study	Comparability of cohorts	Assessment of outcome	Follow-up long enough	Adequacy of follow-up	
Abelha, 2012 [37]	Portugal	Yes		*	*	*		*	*	*	6
Abildstrom, 2004 [48]	Denmark	Yes		*	*	*	**	*	*	*	8
Askar, 2005 [38]	Turkey	Yes		*	*	*		*	*	*	6
Bryson, 2011 [39]	Canada	Yes		*	*	*	***	*	*	*	8
Cai, 2012 [40]	China	Yes	*	*	*	*		*		*	6
Cunningham, 2017 [30]	UK	Yes		*	*	*		*		*	5
Deiner, 2015 [24]	USA	Yes		*	*	*		*	*		4
Guenther, 2013 [41]	Germany	Not mentioned		*	*			*	*	*	5
Heyer, 2005 [49]	USA	Axis I psychiatric disorder patients excluded	*	*	*		*	*		*	6
Klinger, 2018 [25]	USA	Yes		*	*	*		*		*	5
Lelis, 2006 [42]	Brazil	Yes		*	*	*		*		*	5
Leung, 2007 [28]	USA	No		*	*		**	*		*	6
McDonagh, 2010 [43]	USA	Psychiatric illness patients excluded		*	*		**	*	*	*	7
Rentowl, 2004 [44]	UK	Yes		*	*	*	**	*	*	*	8
Robson, 2002 [50]	UK	Not mentioned		*	*			*	*	*	5
Shoair, 2015 [23]	USA	Yes		*	*	*	**	*	*	*	8
Silbert, 2008 [51]	Australia	No		*	*		**	*	*	*	7
Steed, 2001 [45]	UK	Yes		*	*	*	**	*		*	7
Stewart, 2013 [52]	Canada	Not mentioned		*	*			*	*	*	5
Tagarakis, 2007 [46]	Germany	Acute neuropsychiatric disorder patients excluded		*	*			*	*	*	5
Vasunilashorn, 2015 [29]	USA	Yes	*	*	*	*	**	*	*	*	9
Zoll, 2009 [47]	Germany	Yes		*	*	*	**	*	*	*	8

### Association of APOE4 and POCD within 1 week postoperatively

**Fig 2:**
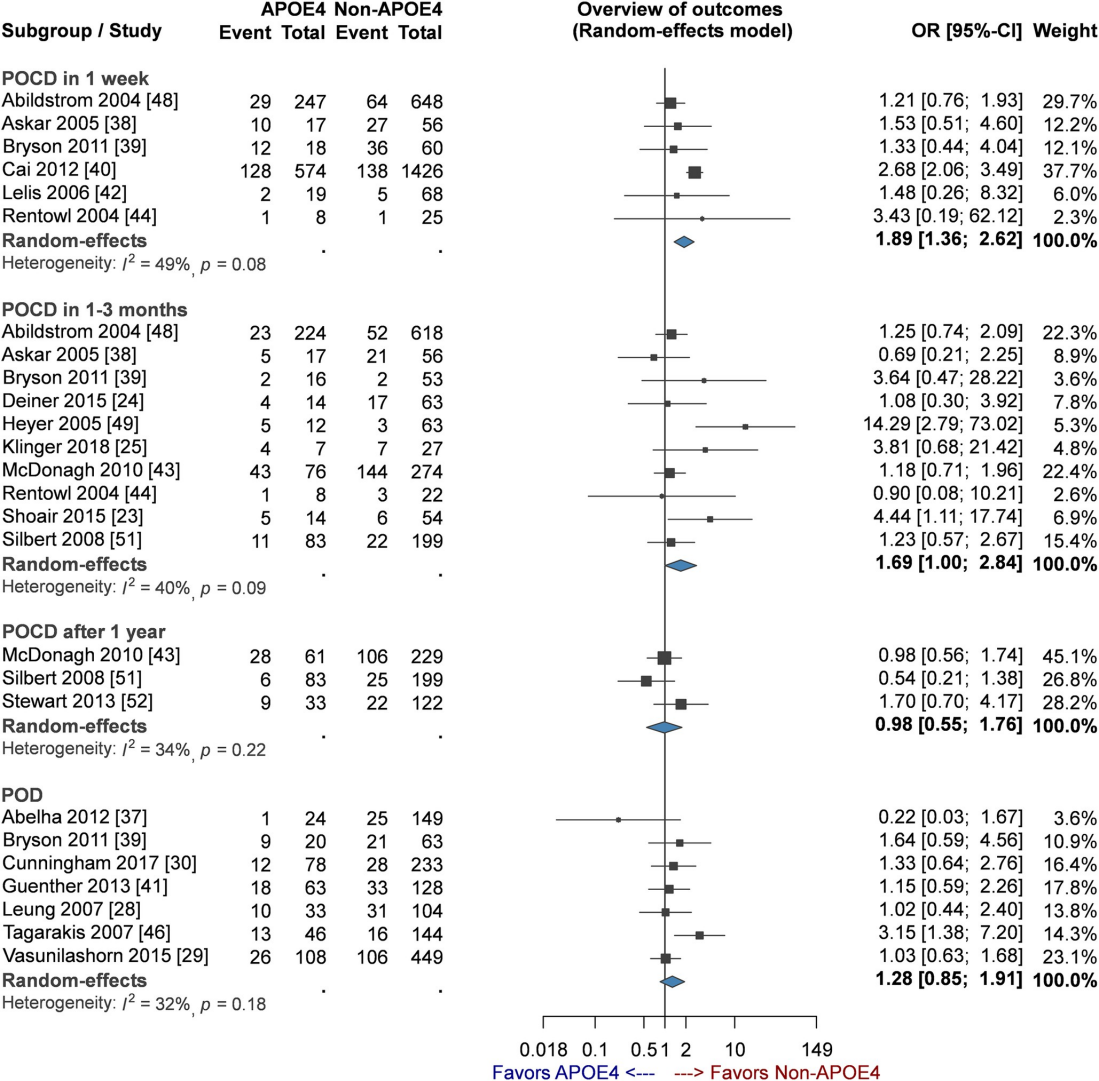
Forest plot of comparison of the risks of POCD or POD in patients with or without ApoE4. Abbreviations: POCD, postoperative cognitive dysfunction; POD, postoperative delirium.

**Fig 3:**
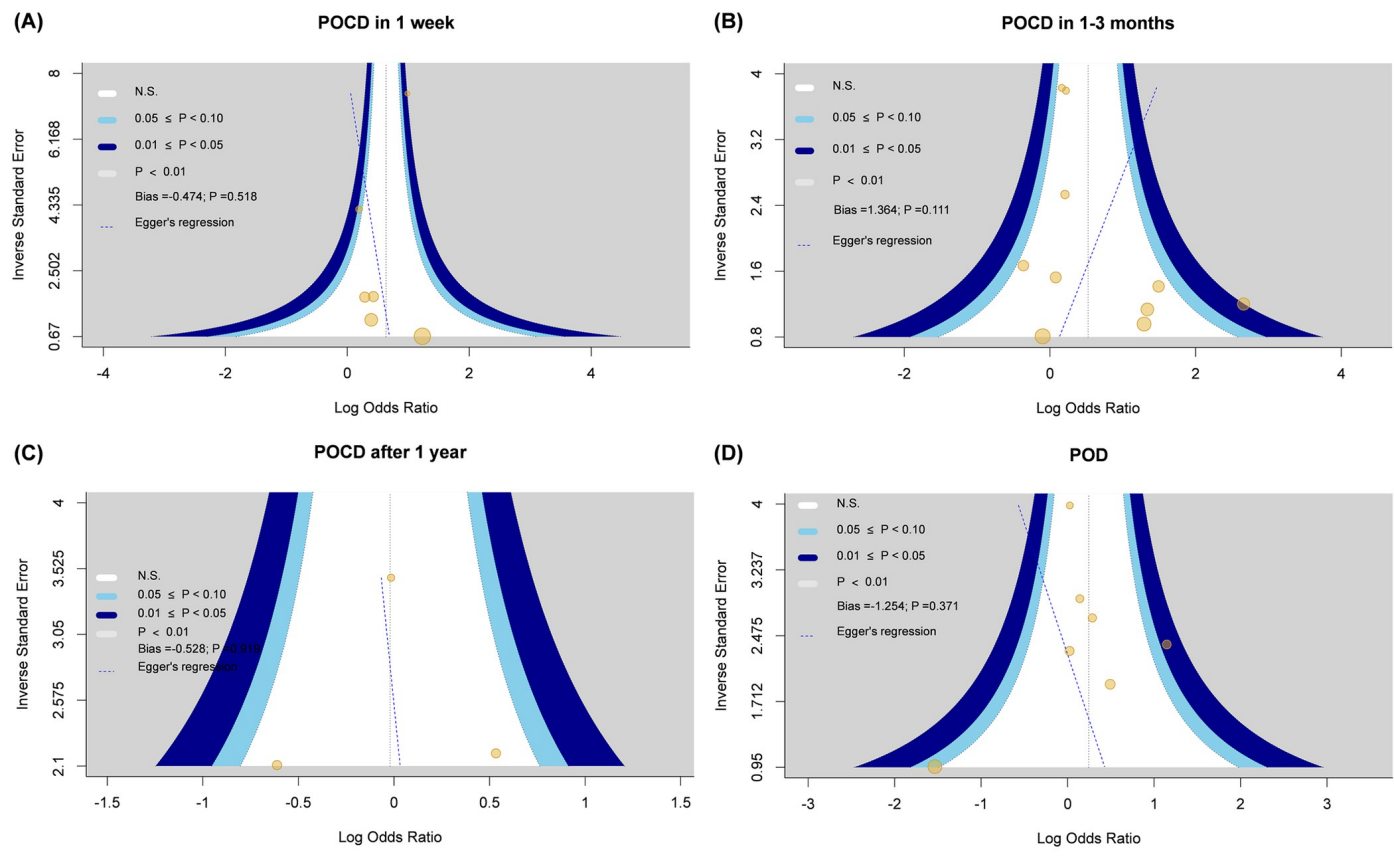
Small-study effect test for POCD or POD. (a) Small-study effect test of POCD at 1 week. (b) Small-study effect test of POCD at 1–3 months. (c) Small-study effect test of POCD after 1 year. (d) Small-study effect test of POD. Abbreviations: POCD, postoperative cognitive dysfunction; POD, postoperative delirium.

Fig 2 (2a. POCD in 1 week), six studies [38–40, 42, 44, 48] including 3,166 participants reported ORs for APOE4 in association with POCD in 1 week. The pooled OR estimate was 1.89 (95% CI: 1.36 to 2.62, p < 0.01) in the random-effects model. Moderate heterogeneity was observed among included studies (*I*^2^ = 49%, p = 0.08), and the likelihood of small-study effect was low (p = 0.52; Fig 3A). The findings of sensitivity analysis showed that heterogeneity among included studies decreased (*I*^2^ = 0%, p = 0.62) when prospective studies were pooled only. This leads to the increase of OR from 1.77 to 2.49 (95% CI: 1.95–3.19; Table 3) (see S1 Fig, which illustrates the sensitivity analyses: effect of different research characteristics on POCD or POD with ApoE4 carriers). However, the results of the other sensitivity analysis demonstrated heterogeneity in the outcomes of risk after excluding patients receiving cardiovascular surgery. To minimize random errors and strengthen the conclusiveness of the results, TSA was applied. It clearly showed that the cumulative Z-curve (z score 3.83) crossed the upper monitoring boundary of TSA (alpha-spending adjusted z score 3.322) for harm (TSA adjusted 95% CI: 1.05 to 3.39; 3166 patients, six trials) establishing firm evidence of the positive result; however, the RIS of 9,095 patients was nearby but not reached (Table 4) (see S2 Fig, which illustrates the trial sequential analysis of the incidence of POCD or POD).

**Table 3:** Sensitivity analyses: Effect of different research characteristics on POCD or POD with ApoE4 carriers.

	POCD at 1 week					POCD at 1–3 months					POD				
Limitations excluded	No. of studies	OR	95% CI	*I* ^2^	*p*	No. of studies	OR	95% CI	*I* ^2^	*p*	No. of studies	OR	95% CI	*I* ^2^	*p*
**Overall**	6	1.89	1.36 to 2.62	49%	<0.01	10	1.69	1.00 to 2.84	40%	0.049	7	1.28	0.85 to 1.91	32%	0.234
**Study design**															
Retrospective study [24, 37, 48, 49, 51]	5	2.49	1.95 to 3.19	0%	<0.01	6	1.56	0.86 to 2.85	26%	0.143	6	1.32	0.96 to 1.83	16%	0.088
**Surgery type**															
Cardiovascular surgery [25, 38, 39, 42, 49, 51]	3	1.93	1.10 to 3.36	76%	0.021	5	1.29	0.92 to 1.8	0%	0.138	6	1.23	0.76 to 2.00	41%	0.401
**Participants**															
Cognitive impairment included [43, 49, 51]	6	1.89	1.36 to 2.62	49%	<0.01	7	1.45	0.91 to 2.32	10%	0.118	7	1.28	0.85 to 1.91	32%	0.234
**Ethnicity**															
Portugal [37]	6	1.89	1.36 to 2.62	49%	<0.01	10	1.69	1.00 to 2.84	40%	0.049	6	1.32	0.96 to 1.83	16%	0.088

NA, not applicable; OR, odd ratio; POCD, postoperative cognitive dysfunctions; POD, postoperative delirium.

**Table 4:** Trial sequential analysis of the incidence of POCD or POD^a^.

Outcomes	No. of studies	Actual sample size	Event proportion in intervention arm (%)	Event proportion in control arm (%)	D^2^ (%)	RIS (sample size)	Z-curve crossed the conventional boundary^b^	TSA adjusted OR	TSA adjusted 95% CI
POCD in 1week	6	3,166	26.38	18.2	56	9,095	Yes	1.89	1.05–3.39
POCD in 1–3 months	10	1,900	23.21	16.1	71	20,516	No	1.69	0.52–5.45
POCD after 1year	3	727	23.34	23.3	48	6,595,285	No	0.98	0.26–3.66
POD	7	1,642	24.92	20.6	52	48,643	No	1.28	0.51–3.17

^a^ TSA calculated an alpha-spending-adjusted required information size by using *α* = 0.05 (two sided) and *β* = 0.20 (power = 80%). The cumulative Z-curve was constructed using a random-effects model.

^b^ The cumulative z-score reached significance by crossing both the conventional boundary.

CI, confidence interval; D^2^, diversity; NA, not applicable; OR, odds ratio; POCD, postoperative cognitive dysfunction; POD, postoperative delirium; RIS, required information size; TSA, trial sequential analysis; Z-curve, cumulative Z-curve.

### POCD within 1–3 months postoperatively

A significant association was observed between APOE4 and POCD in 1–3 months when 10 eligible studies [23–25, 38, 39, 43, 44, 48, 49, 51] and 1900 patients were pooled and analyzed using a random-effects model (OR: 1.69, 95% CI: 1.003–2.839, p = 0.049, *I*^2^ = 40%)(Fig 2B. POCD in 1–3 months). Moderate heterogeneity was observed among included studies (*I*^2^ = 40%; p = 0.09), and the likelihood of small-study effect was not significant (p = 0.11; Fig 3B). In all three sensitivity analyses (Table 3) (see S1 Fig, which illustrates the sensitivity analyses: effect of different research characteristics on POCD or POD with ApoE4 carriers), association was non-significant in prospective studies (OR: 1.56, 95% CI: 0.86–2.85), non-cardiovascular surgery (OR: 1.29, 95% CI: 0.92–1.80), or exclusion of patients with cognitive impairment (OR: 1.45, 95% CI: 0.91–2.30). Although the cumulative Z-curve (z score 1.97) trended to be downward in TSA, it did not cross the trial sequential significance boundary (alpha-spending adjusted z score 6.44) (Table 4) (see S2 Fig, which illustrates the trial sequential analysis of the incidence of POCD and POD). The actual sample size was 1900 which did not reach the RIS of 20,516patients (TSA adjusted 95% CI: 0.52–5.45; 10 trials). Thus, TSA result regarding the association between the APOE4 allele and POCD 1–3 months after surgery was inconclusive and further clinical trials to examine this association are required.

### POCD after 1 year

The association between APOE4 and POCD 1 year after surgery was examined in three cohorts [43, 51, 52], and the pooled data of 727 participants examined using the random-effects model exhibited no significant correlation (OR: 0.98, 95% CI: 0.57–1.75, p = 0.95; Fig 2C). Moderate heterogeneity (*I*^2^ = 34%; p = 0.22) and low small-study effect (p = 0.919; Fig 3C) were observed. In TSA, RIS was not reached, and the cumulative Z-curve (z score -0.07) did not cross the conventional boundary. Moreover, the TSA-adjusted 95% CI could not be calculated because size of the information available being limited regarding POCD after 1 year (actual vs. RIS = 727/6,595,285 = 0.01%; TSA adjusted 95% CI: 0.26–3.66; three trials) (Table 4) (see S2 Fig, which illustrates the trial sequential analysis of the incidence of POCD and POD).

### POD after surgery

In terms of the risk of POD, seven studies [28–30, 37, 39, 41, 46] that had evaluated POD after surgery showed no significant association between APOE4 and POD in the random-effects model (n = 1642; OR: 1.28, 95% CI: 0.85–1.91, p = 0.23; Fig 2D). Between-study heterogeneity was moderate (*I*^2^ = 32%, p = 0.18). Moreover, the likelihood of small-study effect was low (p = 0.18; Fig 3D). The combined findings of all four sensitivity analyses (Table 3) (see S1 Fig, which illustrates the sensitivity analyses: effect of different research characteristics on POCD or POD with ApoE4 carriers) showed no significant association, including prospective studies (OR: 1.32, 95% CI: 0.96–1.83), non-cardiovascular surgery (OR: 1.23, 95% CI: 0.76–2.00), or exclusion of one study conducted in Portugal [33] (OR: 1.32, 95% CI: 0.96–1.83) because of its outlier results (OR: 0.22, 95% CI: 0.03–1.67) from other studies. No study with data regarding POD was excluded due to inclusion of patients with cognitive impairment. The cumulative Z-curve (z score 1.19) in TSA did not cross the conventional boundary before reaching RIS 48,643 (; TSA-adjusted95% CI: 0.51–3.17; seven trials), indicating that the information regarding the association of APOE4 and POD remained inconclusive (Table 4) (see S2 Fig, which illustrates the trial sequential analysis of the incidence of POCD or POD).

## Discussion

This was the first meta-analysis studying the genetic association of POCD or POD in surgical patients carrying at least one copy of APOE4 and using TSA to reinforce its possible linkage. The pooled estimates of our meta-analysis indicated that APOE4 allele was a significant genetic risk factor for POCD 1 week after surgery and being further confirmed its validity by TSA. In addition, this was the first meta-analysis to demonstrate APOE4 also was a significant risk factor for medium-term, 1–3 months, POCD after surgery. Meanwhile, we demonstrated that neither POD nor POCD for 1-year period after surgery was genetically associated with APOE4.

The genetic association of APOE4 and medium-term, 1–3 months, POCD after surgery has its important clinical significance. First, high risk patients with APOE4 received healthcare not only during their hospitalization but also should be followed up to three months for the potential occurrence of neurological dysfunction 1–3 months after surgery [5, 7]. Secondly, the medium-term association of APOE4 and POCD adds weight to the importance and necessity of preoperative screening APOE4 allele before the surgery among high-risk or elderly patients to have early alarm for postoperative neurological dysfunction [54]. Thirdly, based on the positive screening results for the APOE4 allele among high-risk patients, the potential implementation of preventive strategies for POCD among these patients could layout in advance and should not be under-estimated.

Trial sequential analysis (TSA) was applied in this study to further evaluate the power of association of APOE4 allele with postoperative delirium (POD) or short- and medium-term postoperative cognitive dysfunction (POCD). Although there might exist statistical minor discrepancy [55–60] between the conclusion derived from the traditional meta-analysis and TSA, esp. the required number of participants or trials included was not reached [36]. Although the systematic review is academically acknowledged at the top of the generally recognized hierarchy of evidence which represents that the systematic review is considered the most reliable source level of evidence. Researchers consider whether an intervention could be accepted and applied in clinical scenarios although further trials were still needed [61–63]. However, considering the heterogeneity of the outcome (POCD and POD are very different entities) and types of the included studies (cardiovascular surgery and other surgical scenarios), it might downgrade the quality of the evidence as well as the recommendation based on the encountered association.

We have identified another seven studies that examined whether APOE4 was associated with cognitive decline more than 1 year in patients undergoing surgery for extensive interest [16, 55–60]. Two studies revealed a significant association between APOE4 and long-term postoperative cognitive decline [57, 58], but another one study [60] showed no association. Selnes et al. [59] found no significant cognitive decline in patients evaluated 3 years after coronary artery bypass graft surgery, whereas Bartels et al. [55] identified a considerable impact 5 years after cardiac surgery, but not for non-cardiac surgery. Dokkedal et al. [56] reported similar significance in cross-sectional analysis, but not in longitudinal analysis. Furthermore, Schenning et al. [16] indicated that the effect of APOE4 was stronger in men than women. However, meta-analysis for above studies was non-achievable due to extraordinary heterogeneity existed between studies.

We performed sensitivity analyses according to the surgery types for the association of PAOE4 and POD, POCD at 1 week, or POCD at 1–3 months postoperatively. Because surgical trauma *per se* and/or cardiopulmonary bypass during cardiac surgery might cause inflammation. When studies of patients receiving cardiovascular surgery were excluded, none of the POCD or POD groups above were found to be significantly associated with APOE4. Only POCD at 1–3 months after surgery exhibited a lower *I*^2^ value, indicating that the surgery type was not likely to cause heterogeneity in impact for 1–3 months period. Evered et al. [64] found that the incidence of POCD was independent to the types of surgery except in older patients 1 week after coronary artery bypass graft surgery. We did not perform sensitivity analysis about various treatment modalities of different anesthetic technique or agent. However, a recent recommendation study [65] indicated the absence of adequate evidence showing that specific anesthetic techniques or agents would increase the risk of POCD. In contrast, Miller D et al. showed that propofol-based total intravenous anesthesia might reduce the risk of POCD compared with inhalational agents, but the evidence had low certainty [66].

The findings of our sensitivity analyses provided new insights by re-analyzing the risks of POD or POCD at 1–3 months by excluding studies that included participants with cognitive impairment [28, 41, 43, 46, 49, 51] and excluding retrospective studies for POCD 1 week and 1–3 months after surgery. Furthermore, we examined the risk of POD by excluding one study conducted in Portugal [37] because we found it had marked heterogeneity from other studies such as lower APOE4 carrier incidence (13.9%) compared with other studies. Abondio et al. [67] indicated that Portuguese individuals have one of the lowest frequencies of APOE4 (5% to 10%). In addition, studies have indicated that APOE4 exerted a weaker effect on cognitive impairment in Hispanics [21, 68]. Moreover, the Mediterranean diet, which is named for the traditional diet in the Mediterranean area, could prevent cognitive impairment [69]. However, the results of our sensitivity analysis revealed a significant association of POCD with APOE4 only at 1 week or discharge when we excluded retrospective studies. Whereas, sensitivity analyses in the other aspects, did not recruit enough number of studies to obtain significant results.

### Potential mechanisms

Several mechanisms might be involved with the association of APOE4 and POCD. Neuro-inflammation caused by surgical trauma inducing increases in inflammatory cytokines, microglial activation and blood–brain barrier (BBB) disruption [17, 70, 71] might lead to POCD. These effects can subsequently cause synaptic injury and neuronal death. In addition, decreased cerebral blood flow also plays a vital role in POCD. Different types of anesthetics, such as intravenous agent, propofol, provided its neuroprotective property of anti-inflammation and reduced the incidence of POCD when compared with the inhalational anesthetics [6, 15].

APOE4 may be involved in the development of POCD in several mechanisms. APOE4 with increasing age was reported to be associated with decreased cerebral blood flow [72], and this association can predispose developing POCD. In addition, APOE4 reduces amyloid-β clearance [73]. Deposition of amyloid-β plays an essential role in AD by activating microglial cells that release cytokines, thus resulting in neuro-inflammation. These two findings proposed the correlation of amyloid-β deposition, the silent AD pathology in APOE4 carriers, with POCD [70]. Moreover, APOE4 was associated with blood-brain-barrier disruption in AD [73]. The damage in blood-brain-barrier may also be crucial in POCD. Considering that APOE4 was significantly related to both POCD and AD [74]. which implied that POCD and AD might share a common pathology [17]. To support this hypothesis, one recent study found that APOE4 knock-in mice had increased amyloid-β deposition in the hippocampal CA3 region and longer mean escape latencies in the Morris water maze test compared with non-knock-in mice 1 week after abdominal surgery [75].

Neuro-inflammation is believed to be involved in the development of either POD or POCD [17, 20], and they might have similar risk factors including old age, depression, and poor baseline cognitive function. However, based on this meta-analysis, we proposed that APOE4 might play a role in POCD instead of POD, implying different mechanisms involved. Indeed, one view indicated that POCD and POD are dissimilar in a few aspects [76]. First, POCD mainly affects executive or memory function, whereas POD involves different domains of mental status such as inattention, disorientation, and consciousness disturbance [3]. Second, the peak incidence of POCD was from the first to third months, while POD was from the first to third days after surgery. In addition, Daiello et al. [77] reported that POD was related to POCD at the first but not the second or sixth month after major non-cardiac surgery, suggesting a distinction between them. Future studies are needed how POD and POCD could be induced differently in the brain and how APOE4 may modulate these two pathologies separately.

### Strengths and limitations

This study has several advantages. We screened 1255 studies from six databases by using numerous keywords linked to APOE4, POD, or POCD. The numbers of studies, databases, and keywords were all larger than those of the previous meta-analysis [22]. Moreover, we included more studies comparing POD and POCD under the effect of APOE4. We examined any secondary outcome associated with postoperative neurocognitive function, thus obtaining a more comprehensive picture of the association between APOE4 and cognitive complications. The assessment of study quality performed using the Newcastle–Ottawa Scale (NOS) indicated that all our studies included with moderate quality. Moreover, TSA exhibited a significant association providing more strength in evidence about APOE4-POCD relationship within one week after surgery.

Several limitations that should be considered in this study. The definition and assessment methodology for postoperative cognitive alterations varied. Recently, a consensus-working group published recommendations from a panel of specialists suggesting that cognitive assessments on POCD should be distinguished into delayed cognitive recovery (DCR), ie, evaluations up to 30 days postoperative, and postoperative neurocognitive disorder (pNCD), ie, assessments performed between 30 days and 12 months after surgery and recommended treating postoperative delirium as a totally different entity [6]. However, prospective or retrospective studies recruited in this study based on various neuropsychological tests are still valuable and being different from the definition suggested nowadays become the first limitation of our study. Secondly, participants in our study were mostly from the older population; thus, we could not determine whether the effects are the same in middle age or young patients. Furthermore, the minimum SD or Z-score change in cognitive tests required for diagnosing POCD varied from approximately 1 to 2. Although we have performed analysis by using continuous variables to overcome this issue but the data was not enough for further conclusion. In addition, the significant relation between APOE4 and POCD in 1 week or at discharge was contributed by one study with a large sample size [40] which was analyzed at one day instead of one week. More relevant studies in the future are required to confirm our results because TSA showed that the number of participants was below than expected. The discrepancy of clinical incidence of postoperative cognitive dysfunction among non-cardiac and cardiac/vascular surgery is always a major concern when pooling them together in the research of meta-analysis. We have treated data with evidence selection within individual study and sensitivity analysis across the studies to minimize clinical heterogeneity. The association of APOE 4 and either types of surgery were still to be verified due to the case number recruited was limited and further studies to have more case number recruited with recent nomenclature in individual type of surgery is needed.

### Future directions

Because we showed that the presence of APOE4 allele could increase the risk of POCD with not only the short-term but also the medium-term impact, preventive measures should be implemented for patients carrying APOE4. Therefore, a low-cost, rapid, and convenient genetic screen test could be developed for detecting the expression of APOE4 before surgery. A recent review [54] discussed the accessibility of using microarray to determine APOE4 mRNA rapidly and efficiently. Veiga et al. [78] described a novel latex-enhanced immune-turbidimetric blood assay for low-cost, fast, and easy APOE4 genotyping. In addition, Kushioka et al. [79] found that the use of saliva-derived DNA for APOE4 determination, which was non-invasive and more convenient than obtaining blood samples. Calero et al. [80] reported a low-cost, rapid, and convenient detection method using APOE–polystyrene binding interaction. This method was potentially applicable using various biological fluids, including urine or saliva with the advantage that informed consent for DNA isolation was not required. Moreover, various genes other than APOE4 were found to be related to postoperative complications. Dopamine receptor D2 gene (DRD2), solute carrier family 6 member 3 (SLC6A3) and nuclear receptor family 3 group C member 1 (NR3C1) were associated with POD [27, 81]. In the future, there could be an one-for-all gadget rapidly detecting all those genetic expressions simultaneously which was powerful and convenient for POCD prevention. As long as knowing who was at increased genetic risk, several perioperative strategies for cognitive protection could be planned. Administering pre-medications to surgical patients, including cyclooxygenase 2 isozyme (COX-2) inhibitors, statins or dexmedetomidine, could reduce POCD incidence [14]. Oral carbohydrate preloading in the preoperative period could lower neuroendocrine stress response, which attenuates POCD [82].

Intraoperatively, using bi-spectral index to guide depth of anesthesia, maintaining higher mean arterial pressure for cerebral perfusion and avoiding use of medicine with potential negative neurological effects may cause less damage to postoperative cognitive function [83]. After surgery, a peaceful environment with family accompaniment would be beneficial [82]. Eventually, the use of an efficient detection modality can help prevent postoperative cognitive complications in patients and improve their quality of life.

## Conclusion

Findings suggested that APOE-4 was associated with the occurrence of POCD in short-term, the first week and medium-term, and 1–3 months after surgery among surgical patients, but not with POD. Trial sequential analysis showed that the required information size had not reached, and there was insufficient evidence to reach a conclusion. To substantiate the findings, future well designed large case-control studies in this field are recommended.

## Supporting information

S1 Checklist (PDF)

S1 File Search strategy for all electronic databases. (PDF)

S1 Table Details of thirty-three full-text studies excluded. (PDF)

S1 Fig Sensitivity analyses: Effect of different research characteristics on POCD or POD with ApoE4 carriers. (PDF)

S2 Fig Trial sequential analysis of the incidence of POCD or POD. (PDF)
