# Supplementary material for: Apolipoprotein E4 allele is genetically associated with risk of the short- and medium-term postoperative cognitive dysfunction: A meta-analysis and trial sequential analysis
Source: PLoS One. 2023 Feb 24;18(2):e0282214. doi: 10.1371/journal.pone.0282214 (PMC9955600; doi:10.1371/journal.pone.0282214)
Supplement: S1 File — (PDF) [file pone.0282214.s002.pdf]

S1. Search strategy for all electronic databases.

- **PubMed**

#1	(((('acute delirium' OR 'delier' OR 'delire' OR 'deliria' OR 'delirious state' OR 'delirious syndrome' OR 'delirium acutum' OR 'confusion' OR 'hallucinations' OR 'inattentiveness' OR 'disorientation' OR 'illusions' OR 'agitation' OR delirium OR 'postoperative delirium') OR ('acute delirium'[Text Word] OR 'delier'[Text Word] OR 'delire'[Text Word] OR 'deliria'[Text Word] OR 'delirious state'[Text Word] OR 'delirious syndrome'[Text Word] OR 'delirium acutum'[Text Word] OR 'confusion'[Text Word] OR 'hallucinations'[Text Word] OR 'inattentiveness'[Text Word] OR 'disorientation'[Text Word] OR 'illusions'[Text Word] OR 'agitation'[Text Word] OR delirium[Text Word] OR 'postoperative delirium'[Text Word])) OR ('acute delirium' OR 'delier' OR 'delire' OR 'deliria' OR 'delirious state' OR 'delirious syndrome' OR 'delirium acutum' OR 'confusion' OR 'hallucinations' OR 'inattentiveness' OR 'disorientation' OR 'illusions' OR 'agitation' OR delirium OR 'postoperative delirium'[MeSH Terms])) OR (("Postoperative Cognitive Complications"[Mesh]) OR ("postoperative cognitive complications" OR POCD) OR ("postoperative cognitive complications"[Text Word] OR POCD[Text Word]) OR ("postoperative cognitive complications" OR POCD[MeSH Terms]) OR ("postoperative cognitive dysfunction" OR POCD OR "postoperative cognitive dysfunctions") OR ("postoperative cognitive dysfunction"[Text Word] OR POCD[Text Word] OR "postoperative cognitive dysfunctions"[Text Word]) OR ("postoperative cognitive dysfunction" OR POCD OR "postoperative cognitive dysfunctions"[MeSH Terms]) OR ('postoperative cognitive decline') OR ('postoperative cognitive decline'[Text Word]) OR ('postoperative cognitive decline'[MeSH Terms]))) AND (('apoe' OR 'apoe4' OR 'apo e' OR 'apo e4' OR 'apo E4' OR 'apolipoprotein e' OR 'apolipoprotein e4') OR ((('apoe'[Text Word] OR 'apoe4'[Text Word] OR 'apo e'[Text Word] OR 'apo e4'[Text Word] OR 'apolipoprotein e'[Text Word] OR 'apolipoprotein e4'[Text Word]) OR ('apoe' OR 'apoe4' OR 'apo e' OR 'apo e4' OR 'apolipoprotein e' OR 'apolipoprotein e4'[MeSH Terms])) OR (('apolipoprotein e4'[Text Word] OR 'apo E4'[Text Word]) OR ('apolipoprotein e4' OR 'apo E4'[MeSH Terms]))))

- **EMBASE**

#8	#2 AND #7
#7	#1 OR #3 OR #4 OR #5 OR #6
#6	'postoperative cognitive decline'
#5	'postoperative cognitive complications'/exp
#4	'postoperative cognitive dysfunction'/exp
#3	'postoperative cognitive dysfunction'/exp OR pocd
#2	'apoe' OR 'apoe4' OR 'apo e' OR 'apo e4' OR 'apolipoprotein e' OR 'apolipoprotein e4'
#1	'acute delirium'/exp OR 'acute delirium' OR 'delier'/exp OR 'delier' OR 'delire'/exp OR 'delire' OR 'deliria'/exp OR 'deliria' OR 'delirious state'/exp OR 'delirious state' OR 'delirious syndrome'/exp OR 'delirious syndrome' OR 'delirium acutum'/exp OR 'delirium acutum' OR 'confusion'/exp OR 'confusion' OR 'hallucinations'/exp OR 'hallucinations' OR 'inattentiveness' OR 'disorientation'/exp OR 'disorientation' OR 'illusions'/exp OR 'illusions' OR 'agitation'/exp OR 'agitation' OR 'delirium'/exp OR delirium OR 'postoperative delirium'/exp OR 'postoperative delirium'

- **Cochrane**

#1	(“acute delirium” OR “delier” OR “delire” OR “deliria” OR “delirious state” OR “delirious syndrome” OR “delirium acutum” OR “confusion” OR “hallucinations” OR “inattentiveness” OR “disorientation” OR “illusions” OR “agitation” OR delirium OR “postoperative delirium”):ti,ab,kw OR (“acute delirium” OR “delier” OR “delire” OR “deliria” OR “delirious state” OR “delirious syndrome” OR “delirium acutum” OR “confusion” OR “hallucinations” OR “inattentiveness” OR “disorientation” OR “illusions” OR “agitation” OR delirium OR “postoperative delirium”) (Word variations have been searched)

#2	MeSH descriptor: [Delirium] explode all trees
#3	(“postoperative cognitive dysfunction” OR POCD OR “postoperative cognitive complications” OR “postoperative cognitive decline”):ti,ab,kw OR (“postoperative cognitive dysfunction” OR POCD OR “postoperative cognitive complications” OR “postoperative cognitive decline”) (Word variations have been searched)
#4	MeSH descriptor: [Postoperative Cognitive Complications] explode all trees
#5	(“apoe” OR “apoe4” OR “apo e” OR “apo e4” OR “apo E4” OR “apolipoprotein e” OR “apolipoprotein e4”):ti,ab,kw OR (“apoe” OR “apoe4” OR “apo e” OR “apo e4” OR “apo E4” OR “apolipoprotein e” OR “apolipoprotein e4”) (Word variations have been searched)
#6	MeSH descriptor: [Apolipoprotein E4] explode all trees
#7	#1 OR #2 OR #3 OR #4
#8	#5 OR #6
#9	#7 AND #8
#10	(#1 OR #2 OR #3 OR #4) AND (#7 AND #8)

- **CINAHL**

S4	S1 AND S2
S3	TX (‘apoe’ OR ‘apoe4’ OR ‘apo e’ OR ‘apo e4’ OR ‘apo E4’ OR ‘apolipoprotein e’ OR ‘apolipoprotein e4’) OR TI (‘apoe’ OR ‘apoe4’ OR ‘apo e’ OR ‘apo e4’ OR ‘apo E4’ OR ‘apolipoprotein e’ OR ‘apolipoprotein e4’) OR MJ (‘apoe’ OR ‘apoe4’ OR ‘apo e’ OR ‘apo e4’ OR ‘apo E4’ OR ‘apolipoprotein e’ OR ‘apolipoprotein e4’) OR SU (‘apoe’ OR ‘apoe4’ OR ‘apo e’ OR ‘apo e4’ OR ‘apo E4’ OR ‘apolipoprotein e’ OR ‘apolipoprotein e4’) OR MW (‘apoe’ OR ‘apoe4’ OR ‘apo e’ OR ‘apo e4’ OR ‘apo E4’ OR ‘apolipoprotein e’ OR ‘apolipoprotein e4’) OR AB ((‘apoe’ OR ‘apoe4’ OR ‘apo e’ OR ‘apo e4’ OR ‘apo E4’ OR ‘apolipoprotein e’ OR ‘apolipoprotein e4’) OR (‘apoe’ OR ‘apoe4’ OR ‘apo e’ OR ‘apo e4’ OR ‘apo E4’ OR ‘apolipoprotein e’ OR ‘apolipoprotein e4’))
S2	TX (‘apoe’ OR ‘apoe4’ OR ‘apo e’ OR ‘apo e4’ OR ‘apo E4’ OR ‘apolipoprotein e’ OR ‘apolipoprotein e4’) OR TI (‘apoe’ OR ‘apoe4’ OR

	‘apo e’ OR ‘apo e4’ OR ‘apo E4’ OR ‘apolipoprotein e’ OR ‘apolipoprotein e4’) OR MJ (‘apoe’ OR ‘apoe4’ OR ‘apo e’ OR ‘apo e4’ OR ‘apo E4’ OR ‘apolipoprotein e’ OR ‘apolipoprotein e4’) OR SU (‘apoe’ OR ‘apoe4’ OR ‘apo e’ OR ‘apo e4’ OR ‘apo E4’ OR ‘apolipoprotein e’ OR ‘apolipoprotein e4’) OR MW (‘apoe’ OR ‘apoe4’ OR ‘apo e’ OR ‘apo e4’ OR ‘apo E4’ OR ‘apolipoprotein e’ OR ‘apolipoprotein e4’) OR AB (((‘apoe’ OR ‘apoe4’ OR ‘apo e’ OR ‘apo e4’ OR ‘apo E4’ OR ‘apolipoprotein e’ OR ‘apolipoprotein e4’)) OR ((‘apoe’ OR ‘apoe4’ OR ‘apo e’ OR ‘apo e4’ OR ‘apo E4’ OR ‘apolipoprotein e’ OR ‘apolipoprotein e4’)))
S1	TX (‘acute delirium’ OR ‘delier’ OR ‘delire’ OR ‘deliria’ OR ‘delirious state’ OR ‘delirious syndrome’ OR ‘delirium acutum’ OR ‘confusion’ OR ‘hallucinations’ OR ‘inattentiveness’ OR ‘disorientation’ OR ‘illusions’ OR ‘agitation’ OR delirium OR ‘postoperative delirium’ OR ‘postoperative cognitive dysfunction’ OR POCD OR ‘postoperative cognitive complications’ OR ‘postoperative cognitive decline’) OR TI (‘acute delirium’ OR ‘delier’ OR ‘delire’ OR ‘deliria’ OR ‘delirious state’ OR ‘delirious syndrome’ OR ‘delirium acutum’ OR ‘confusion’ OR ‘hallucinations’ OR ‘inattentiveness’ OR ‘disorientation’ OR ‘illusions’ OR ‘agitation’ OR delirium OR ‘postoperative delirium’ OR ‘postoperative cognitive dysfunction’ OR POCD OR ‘postoperative cognitive complications’ OR ‘postoperative cognitive decline’) OR MJ (‘acute delirium’ OR ‘delier’ OR ‘delire’ OR ‘deliria’ OR ‘delirious state’ OR ‘delirious syndrome’ OR ‘delirium acutum’ OR ‘confusion’ OR ‘hallucinations’ OR ‘inattentiveness’ OR ‘disorientation’ OR ‘illusions’ OR ‘agitation’ OR delirium OR ‘postoperative delirium’ OR ‘postoperative cognitive dysfunction’ OR POCD OR ‘postoperative cognitive complications’ OR ‘postoperative cognitive decline’) OR SU (‘acute delirium’ OR ‘delier’ OR ‘delire’ OR ‘deliria’ OR ‘delirious state’ OR ‘delirious syndrome’ OR ‘delirium acutum’ OR ‘confusion’ OR ‘hallucinations’ OR ‘inattentiveness’ OR ‘disorientation’ OR ‘illusions’ OR ‘agitation’ OR delirium OR ‘postoperative delirium’ OR ‘postoperative cognitive dysfunction’ OR POCD OR ‘postoperative cognitive complications’ OR ‘postoperative cognitive decline’) OR MW (‘acute delirium’ OR ‘delier’ OR ‘delire’ OR ‘deliria’ OR ‘delirious state’ OR ‘delirious syndrome’ OR ‘delirium acutum’ OR ‘confusion’ OR ‘hallucinations’ OR ‘inattentiveness’ OR ‘disorientation’ OR ‘illusions’ OR ‘agitation’ OR delirium OR ‘postoperative delirium’ OR ‘postoperative cognitive dysfunction’ OR POCD

	OR ‘postoperative cognitive complications’ OR ‘postoperative cognitive decline’) OR AB (((‘acute delirium’ OR ‘delier’ OR ‘delire’ OR ‘deliria’ OR ‘delirious state’ OR ‘delirious syndrome’ OR ‘delirium acutum’ OR ‘confusion’ OR ‘hallucinations’ OR ‘inattentiveness’ OR ‘disorientation’ OR ‘illusions’ OR ‘agitation’ OR delirium OR ‘postoperative delirium’ OR ‘postoperative cognitive dysfunction’ OR POCD OR ‘postoperative cognitive complications’ OR ‘postoperative cognitive decline’)) OR ((‘acute delirium’ OR ‘delier’ OR ‘delire’ OR ‘deliria’ OR ‘delirious state’ OR ‘delirious syndrome’ OR ‘delirium acutum’ OR ‘confusion’ OR ‘hallucinations’ OR ‘inattentiveness’ OR ‘disorientation’ OR ‘illusions’ OR ‘agitation’ OR delirium OR ‘postoperative delirium’ OR ‘postoperative cognitive dysfunction’ OR POCD OR ‘postoperative cognitive complications’ OR ‘postoperative cognitive decline’)))

- **Web of Science**

#1	TS=((“acute delirium” OR “delier” OR “delire” OR “deliria” OR “delirious stat e” OR “delirious syndrome” OR “delirium acutum” OR “confusion” OR “hallu cinations” OR “inattentiveness” OR “disorientation” OR “illusions” OR “agitati on” OR delirium OR “postoperative delirium” OR “postoperative cognitive dys function” OR POCD OR “postoperative cognitive complications” OR “postoper ative cognitive decline”) AND (“apoe” OR “apoe4” OR “apo e” OR “apo e4” O R “apo E4” OR “apolipoprotein e” OR “apolipoprotein e4”)) OR TI=((“acute d elirium” OR “delier” OR “delire” OR “deliria” OR “delirious state” OR “delirio us syndrome” OR “delirium acutum” OR “confusion” OR “hallucinations” OR “inattentiveness” OR “disorientation” OR “illusions” OR “agitation” OR deliriu m OR “postoperative delirium” OR “postoperative cognitive dysfunction” OR P OCD OR “postoperative cognitive complications” OR “postoperative cognitive decline”) AND (“apoe” OR “apoe4” OR “apo e” OR “apo e4” OR “apo E4” OR “apolipoprotein e” OR “apolipoprotein e4”)) OR KP=((“acute delirium” OR “ delier” OR “delire” OR “deliria” OR “delirious state” OR “delirious syndrome” OR “delirium acutum” OR “confusion” OR “hallucinations” OR “inattentivene ss” OR “disorientation” OR “illusions” OR “agitation” OR delirium OR “posto perative delirium” OR “postoperative cognitive dysfunction” OR POCD OR “p ostoperative cognitive complications” OR “postoperative cognitive decline”) A ND (“apoe” OR “apoe4” OR “ap

- **PsycINFO**

S3	S1 AND S2
S2	TX ((‘apoe’ OR ‘apoe4’ OR ‘apo e’ OR ‘apo e4’ OR ‘apo E4’ OR ‘apolipoprotein e’ OR ‘apolipoprotein e4’)) OR TI ((‘apoe’ OR ‘apoe4’ OR ‘apo e’ OR ‘apo e4’ OR ‘apo E4’ OR ‘apolipoprotein e’ OR ‘apolipoprotein e4’)) OR MA ((‘apoe’ OR ‘apoe4’ OR ‘apo e’ OR ‘apo e4’ OR ‘apo E4’ OR ‘apolipoprotein e’ OR ‘apolipoprotein e4’)) OR KW ((‘apoe’ OR ‘apoe4’ OR ‘apo e’ OR ‘apo e4’ OR ‘apo E4’ OR ‘apolipoprotein e’ OR ‘apolipoprotein e4’))
S1	TX ((‘acute delirium’ OR ‘delier’ OR ‘delire’ OR ‘deliria’ OR ‘delirious state’ OR ‘delirious syndrome’ OR ‘delirium acutum’ OR ‘confusion’ OR ‘hallucinations’ OR ‘inattentiveness’ OR ‘disorientation’ OR ‘illusions’ OR ‘agitation’ OR delirium OR ‘postoperative delirium’ OR ‘postoperative cognitive dysfunction’ OR POCD OR ‘postoperative cognitive complications’ OR ‘postoperative cognitive decline’)) OR TI ((‘acute delirium’ OR ‘delier’ OR ‘delire’ OR ‘deliria’ OR ‘delirious state’ OR ‘delirious syndrome’ OR ‘delirium acutum’ OR ‘confusion’ OR ‘hallucinations’ OR ‘inattentiveness’ OR ‘disorientation’ OR ‘illusions’ OR ‘agitation’ OR delirium OR ‘postoperative delirium’ OR ‘postoperative cognitive dysfunction’ OR POCD OR ‘postoperative cognitive complications’ OR ‘postoperative cognitive decline’)) OR MA ((‘acute delirium’ OR ‘delier’ OR ‘delire’ OR ‘deliria’ OR ‘delirious state’ OR ‘delirious syndrome’ OR ‘delirium acutum’ OR ‘confusion’ OR ‘hallucinations’ OR ‘inattentiveness’ OR ‘disorientation’ OR ‘illusions’ OR ‘agitation’ OR delirium OR ‘postoperative delirium’ OR ‘postoperative cognitive dysfunction’ OR POCD OR ‘postoperative cognitive complications’ OR ‘postoperative cognitive decline’)) OR KW ((‘acute delirium’ OR ‘delier’ OR ‘delire’ OR ‘deliria’ OR ‘delirious state’ OR ‘delirious syndrome’ OR ‘delirium acutum’ OR ‘confusion’ OR ‘hallucinations’ OR ‘inattentiveness’ OR ‘disorientation’ OR ‘illusions’ OR ‘agitation’ OR delirium OR ‘postoperative delirium’ OR ‘postoperative cognitive dysfunction’ OR POCD OR ‘postoperative cognitive complications’ OR ‘postoperative cognitive decline’))
