# Supplementary material for: Apolipoprotein E4 allele is genetically associated with risk of the short- and medium-term postoperative cognitive dysfunction: A meta-analysis and trial sequential analysis
Source: PLoS One. 2023 Feb 24;18(2):e0282214. doi: 10.1371/journal.pone.0282214 (PMC9955600; doi:10.1371/journal.pone.0282214)
Supplement: S1 Table — (PDF) [file pone.0282214.s003.pdf]

S2 Table. Details of thirty-three full-text studies excluded.

Study	Excluded reason
Adamis et al., 2009[1]<br>Adamis et al., 2014[2]<br>Adamis, Treloar, Gregson, Macdonald, & Martin, 2011[3]<br>Adamis et al., 2007[4]<br>Alexander et al., 2014[5]<br>Bowman et al., 2019[6]<br>Ely et al., 2007[7]<br>Oldenbeuving, de Kort, Kappelle, van Duijn, & Roks, 2013[8]	Patients not receiving surgery
Bromander et al., 2012[9]<br>Mathew et al., 2007[10]<br>Nellis et al., 2018[11]<br>Newman et al., 2001[12]	Lacking the APOE4 allele
Ancelin et al., 2010[13]<br>Chapin et al., 2008[14]<br>Fjell et al., 2018[15]<br>Fu et al., 2010[16]<br>Gaynor et al., 2009[17]<br>Zhang, Wang, Yu, Zhan, & Chen, 2008[18]	Had different research aims/outcomes from this study
Bartels et al., 2015[19]<br>Brown et al., 2015[20]<br>Dokkedal et al., 2020[21]<br>Patel, Lunn, Smith, Lehmann, & Dorrington, 2016[22]<br>Schenning, Murchison, Mattek, Kaye, & Quinn, 2019[23]<br>Schenning et al., 2016[24]<br>Selnes et al., 2007[25]<br>Sprung et al., 2016[26]	Studying only long-term POCD records
Ding et al., 2021[27]<br>Tardiff et al., 1997[28]<br>Yocum et al., 2009[29]	Lacking detailed data
Cunningham et al., 2019[30]<br>Vasunilashorn et al., 2015[31]<br>Vasunilashorn et al., 2020[32]	Studies reporting data from the same cohort
Butterbrod et al., 2021[33]	Patients undergoing neurological surgery

1. Adamis D, Lunn M, Martin FC, Treloar A, Gregson N, Hamilton G, et al. Cytokines and IGF-I in delirious and non-delirious acutely ill older medical inpatients. Age and ageing. 2009;38(3):326-32; discussion 251. Epub 2009/03/10. doi: 10.1093/ageing/afp014. PubMed PMID: 19269948.
2. Adamis D, Meagher D, Treloar A, Dunne C, Larvin M, Martin FC, et al. Phenomenological and biological correlates of improved cognitive function in hospitalized elderly medical inpatients. Archives of gerontology and geriatrics. 2014;59(3):593-8. Epub 2014/09/06. doi: 10.1016/j.archger.2014.08.007. PubMed PMID: 25189345.
3. Adamis D, Treloar A, Gregson N, Macdonald AJ, Martin FC. Delirium and the functional recovery of older medical inpatients after acute illness: the significance of biological factors. Archives of gerontology and geriatrics. 2011;52(3):276-80. Epub 2010/05/18. doi: 10.1016/j.archger.2010.04.006. PubMed PMID: 20471115.
4. Adamis D, Treloar A, Martin FC, Gregson N, Hamilton G, Macdonald AJ. APOE and cytokines as biological markers for recovery of prevalent delirium in elderly medical inpatients. International journal of geriatric psychiatry. 2007;22(7):688-94. Epub 2007/01/05. doi: 10.1002/gps.1732. PubMed PMID: 17203511.
5. Alexander SA, Ren D, Gunn SR, Kochanek PM, Tate J, Ikonomovic M, et al. Interleukin 6 and apolipoprotein E as predictors of acute brain dysfunction and survival in critical care patients. American journal of critical care : an official publication, American Association of Critical-Care Nurses. 2014;23(1):49-57. Epub 2014/01/03. doi: 10.4037/ajcc2014578. PubMed PMID: 24382617;

PubMed Central PMCID: PMCPMC4809634.

6. Bowman K, Jones L, Pilling LC, Delgado J, Kuchel GA, Ferrucci L, et al. Vitamin D levels and risk of delirium: A mendelian randomization study in the UK Biobank. Neurology. 2019;92(12):e1387-e94. Epub 2019/02/17. doi: 10.1212/wnl.0000000000007136. PubMed PMID: 30770424; PubMed Central PMCID: PMCPMC6511096.

7. Ely EW, Girard TD, Shintani AK, Jackson JC, Gordon SM, Thomason JW, et al. Apolipoprotein E4 polymorphism as a genetic predisposition to delirium in critically ill patients. Critical care medicine. 2007;35(1):112-7. Epub 2006/11/30. doi: 10.1097/01.Ccm.0000251925.18961.Ca. PubMed PMID: 17133176.

8. Oldenbeuving AW, de Kort PL, Kappelle LJ, van Duijn CM, Roks G. Delirium in the acute phase after stroke and the role of the apolipoprotein E gene. The American journal of geriatric psychiatry : official journal of the American Association for Geriatric Psychiatry. 2013;21(10):935-7. Epub 2013/08/24. doi: 10.1016/j.jagp.2013.01.068. PubMed PMID: 23969090.

9. Bromander S, Anckarsater R, Kristiansson M, Blennow K, Zetterberg H, Anckarsater H, et al. Changes in serum and cerebrospinal fluid cytokines in response to non-neurological surgery: an observational study. Journal of Neuroinflammation. 2012;9. doi: 10.1186/1742-2094-9-242. PubMed PMID: WOS:000314751100001.

10. Mathew JP, Podgoreanu MV, Grocott HP, White WD, Morris RW, Stafford-Smith M, et al. Genetic Variants in P-Selectin and C-Reactive Protein Influence Susceptibility to Cognitive Decline After

Cardiac Surgery. Journal of the American College of Cardiology. 2007;49(19):1934-42. doi: 10.1016/j.jacc.2007.01.080.

11. Nellis ME, Goel R, Feinstein S, Shahbaz S, Kaur S, Traube C. Association Between Transfusion of RBCs and Subsequent Development of Delirium in Critically Ill Children. Pediatric Critical Care Medicine. 2018;19(10):925-9. doi: 10.1097/PCC.0000000000001675. PubMed PMID: 132358578. Language: English. Entry Date: 20190619. Revision Date: 20191023. Publication Type: journal article.
12. Newman MF, Kirchner JL, Phillips-Bute B, Gaver V, Grocott H, Jones RH, et al. Longitudinal assessment of neurocognitive function after coronary-artery bypass surgery. New England Journal of Medicine. 2001;344(6):395-402. doi: 10.1056/nejm200102083440601. PubMed PMID: WOS:000166800600001.
13. Ancelin ML, de Roquefeuil G, Scali J, Bonnel F, Adam JF, Cheminal JC, et al. Long-term post-operative cognitive decline in the elderly: the effects of anesthesia type, apolipoprotein E genotype, and clinical antecedents. Journal of Alzheimer's disease : JAD. 2010;22 Suppl 3:105-13. Epub 2010/09/23. doi: 10.3233/jad-2010-100807. PubMed PMID: 20858969; PubMed Central PMCID: PMCPMC3078520.
14. Chapin JS, Busch RM, Janigro D, Dougherty M, Tilelli CQ, Lineweaver TT, et al. APOE epsilon4 is associated with postictal confusion in patients with medically refractory temporal lobe epilepsy. Epilepsy research. 2008;81(2-3):220-4. Epub 2008/08/02. doi: 10.1016/j.eplepsyres.2008.05.003.

PubMed PMID: 18672349.

15. Fjell AM, Idland AV, Sala-Llonch R, Watne LO, Borza T, Brækhus A, et al. Neuroinflammation and Tau Interact with Amyloid in Predicting Sleep Problems in Aging Independently of Atrophy. Cerebral cortex (New York, NY : 1991). 2018;28(8):2775-85. Epub 2017/06/29. doi: 10.1093/cercor/bhx157. PubMed PMID: 28655157.
16. Fu YH, Lv RJ, Jin LR, Lu Q, Shao XQ, He JS, et al. Association of apolipoprotein E polymorphisms with temporal lobe epilepsy in a Chinese Han population. Epilepsy research. 2010;91(2-3):253-9. Epub 2010/09/03. doi: 10.1016/j.eplepsyres.2010.07.020. PubMed PMID: 20810250.
17. Gaynor JW, Nord AS, Wernovsky G, Bernbaum J, Solot CB, Burnham N, et al. Apolipoprotein E genotype modifies the risk of behavior problems after infant cardiac surgery. Pediatrics. 2009;124(1):241-50. Epub 2009/07/01. doi: 10.1542/peds.2008-2281. PubMed PMID: 19564306; PubMed Central PMCID: PMCPMC2840402.
18. Zhang SQ, Wang G, Yu W, Zhan H, Chen HW. [Relationship between apolipoprotein e4 allele and emergence agitation in patients undergoing general anesthesia]. Nan fang yi ke da xue xue bao = Journal of Southern Medical University. 2008;28(9):1652-3. Epub 2008/09/30. PubMed PMID: 18819891.
19. Bartels K, Li YJ, Li YW, White WD, Laskowitz DT, Kertai MD, et al. Apolipoprotein epsilon 4 genotype is associated with less improvement in cognitive function five years after cardiac surgery:

a retrospective cohort study. Canadian journal of anaesthesia = Journal canadien d'anesthesie. 2015;62(6):618-26. Epub 2015/03/07. doi: 10.1007/s12630-015-0337-8. PubMed PMID: 25744138; PubMed Central PMCID: PMCPMC4529992.

20. Brown CH, Sharrett AR, Coresh J, Schneider ALC, Alonso A, Knopman DS, et al. Association of hospitalization with long-term cognitive and brain MRI changes in the ARIC cohort. Neurology. 2015;84(14):1443-53. doi: 10.1212/wnl.0000000000001439. PubMed PMID: WOS:000352493700012.

21. Dokkedal U, Wod M, Thinggaard M, Hansen TG, Rasmussen LS, Christensen K, et al. Apolipoprotein E ε4 and cognitive function after surgery in middle-aged and elderly Danish twins. Eur J Anaesthesiol. 2020;37(11):984-91. Epub 2020/07/04. doi: 10.1097/eja.0000000000001250. PubMed PMID: 32618758; PubMed Central PMCID: PMCPMC7680924.

22. Patel D, Lunn AD, Smith AD, Lehmann DJ, Dorrington KL. Cognitive decline in the elderly after surgery and anaesthesia: results from the Oxford Project to Investigate Memory and Ageing (OPTIMA) cohort. Anaesthesia. 2016;71(10):1144-52. doi: 10.1111/anae.13571.

23. Schenning KJ, Murchison CF, Mattek NC, Kaye JA, Quinn JF. Sex and genetic differences in postoperative cognitive dysfunction: a longitudinal cohort analysis. Biology of sex differences. 2019;10(1):14. Epub 2019/03/30. doi: 10.1186/s13293-019-0228-8. PubMed PMID: 30922389; PubMed Central PMCID: PMCPMC6440164.

24. Schenning KJ, Murchison CF, Mattek NC, Silbert LC, Kaye JA, Quinn JF. Surgery is associated

with ventricular enlargement as well as cognitive and functional decline. Alzheimer's & dementia : the journal of the Alzheimer's Association. 2016;12(5):590-7. Epub 2015/11/28. doi: 10.1016/j.jalz.2015.10.004. PubMed PMID: 26610898; PubMed Central PMCID: PMCPMC4861667.

25. Selnes OA, Grega MA, Bailey MM, Pham L, Zeger S, Baumgartner WA, et al. Neurocognitive outcomes 3 years after coronary artery bypass graft surgery: a controlled study. The Annals of thoracic surgery. 2007;84(6):1885-96. Epub 2007/11/27. doi: 10.1016/j.athoracsur.2007.06.054. PubMed PMID: 18036903.

26. Sprung J, Roberts RO, Knopman DS, Olive DM, Gappa JL, Sifuentes VL, et al. Association of Mild Cognitive Impairment With Exposure to General Anesthesia for Surgical and Nonsurgical Procedures: A Population-Based Study. Mayo Clinic proceedings. 2016;91(2):208-17. Epub 2016/01/25. doi: 10.1016/j.mayocp.2015.10.023. PubMed PMID: 26803349; PubMed Central PMCID: PMCPMC4967932.

27. Ding DF, Wang P, Jiang YX, Zhang XP, Shi W, Luo YW. Effects of Apolipoprotein E ε4 allele on early postoperative cognitive dysfunction after anesthesia. Anaesthesist. 2021. Epub 2021/06/19. doi: 10.1007/s00101-021-00972-1. PubMed PMID: 34143234.

28. Tardiff BE, Newman MF, Saunders AM, Strittmatter WJ, Blumenthal JA, White WD, et al. Preliminary report of a genetic basis for cognitive decline after cardiac operations. The Neurologic Outcome Research Group of the Duke Heart Center. The Annals of thoracic surgery.

1997;64(3):715-20. Epub 1997/10/06. doi: 10.1016/s0003-4975(97)00757-1. PubMed PMID: 9307463.

29. Yocum GT, Gaudet JG, Lee SS, Stern Y, Teverbaugh LA, Sciacca RR, et al. Inducible nitric oxide synthase promoter polymorphism affords protection against cognitive dysfunction after carotid endarterectomy. Stroke. 2009;40(5):1597-603. Epub 2009/03/17. doi: 10.1161/strokeaha.108.541177. PubMed PMID: 19286578; PubMed Central PMCID: PMCPMC2674524.

30. Cunningham EL, McGuinness B, McAuley DF, Toombs J, Mawhinney T, O'Brien S, et al. CSF Beta-amyloid 1-42 Concentration Predicts Delirium Following Elective Arthroplasty Surgery in an Observational Cohort Study. Annals of surgery. 2019;269(6):1200-5. Epub 2019/05/15. doi: 10.1097/sla.0000000000002684. PubMed PMID: 31082921.

31. Vasunilashorn SM, Ngo L, Inouye SK, Libermann TA, Jones RN, Alsop DC, et al. Cytokines and Postoperative Delirium in Older Patients Undergoing Major Elective Surgery. The journals of gerontology Series A, Biological sciences and medical sciences. 2015;70(10):1289-95. Epub 2015/07/29. doi: 10.1093/gerona/glv083. PubMed PMID: 26215633; PubMed Central PMCID: PMCPMC4817082.

32. Vasunilashorn SM, Ngo LH, Inouye SK, Fong TG, Jones RN, Dillon ST, et al. Apolipoprotein E genotype and the association between C-reactive protein and postoperative delirium: Importance of gene-protein interactions. Alzheimer's & dementia : the journal of the Alzheimer's Association.

2020;16(3):572-80. Epub 2019/11/26. doi: 10.1016/j.jalz.2019.09.080. PubMed PMID: 31761478; PubMed Central PMCID: PMCPMC7086383.

33. Butterbrod E, Sitskoorn M, Bakker M, Jakobs B, Fleischeuer R, Roijers J, et al. The APOE ε4 allele in relation to pre- and postsurgical cognitive functioning of patients with primary brain tumors. European journal of neurology. 2021;28(5):1665-76. Epub 2020/12/21. doi: 10.1111/ene.14693. PubMed PMID: 33342004; PubMed Central PMCID: PMCPMC8247965.
