# Supplementary material for: Apolipoprotein E4 allele is genetically associated with risk of the short- and medium-term postoperative cognitive dysfunction: A meta-analysis and trial sequential analysis
Source: PLoS One. 2023 Feb 24;18(2):e0282214. doi: 10.1371/journal.pone.0282214 (PMC9955600; doi:10.1371/journal.pone.0282214)

S3 Fig. Sensitivity analyses: Effect of different research characteristics on POCD or POD with ApoE4 carriers.

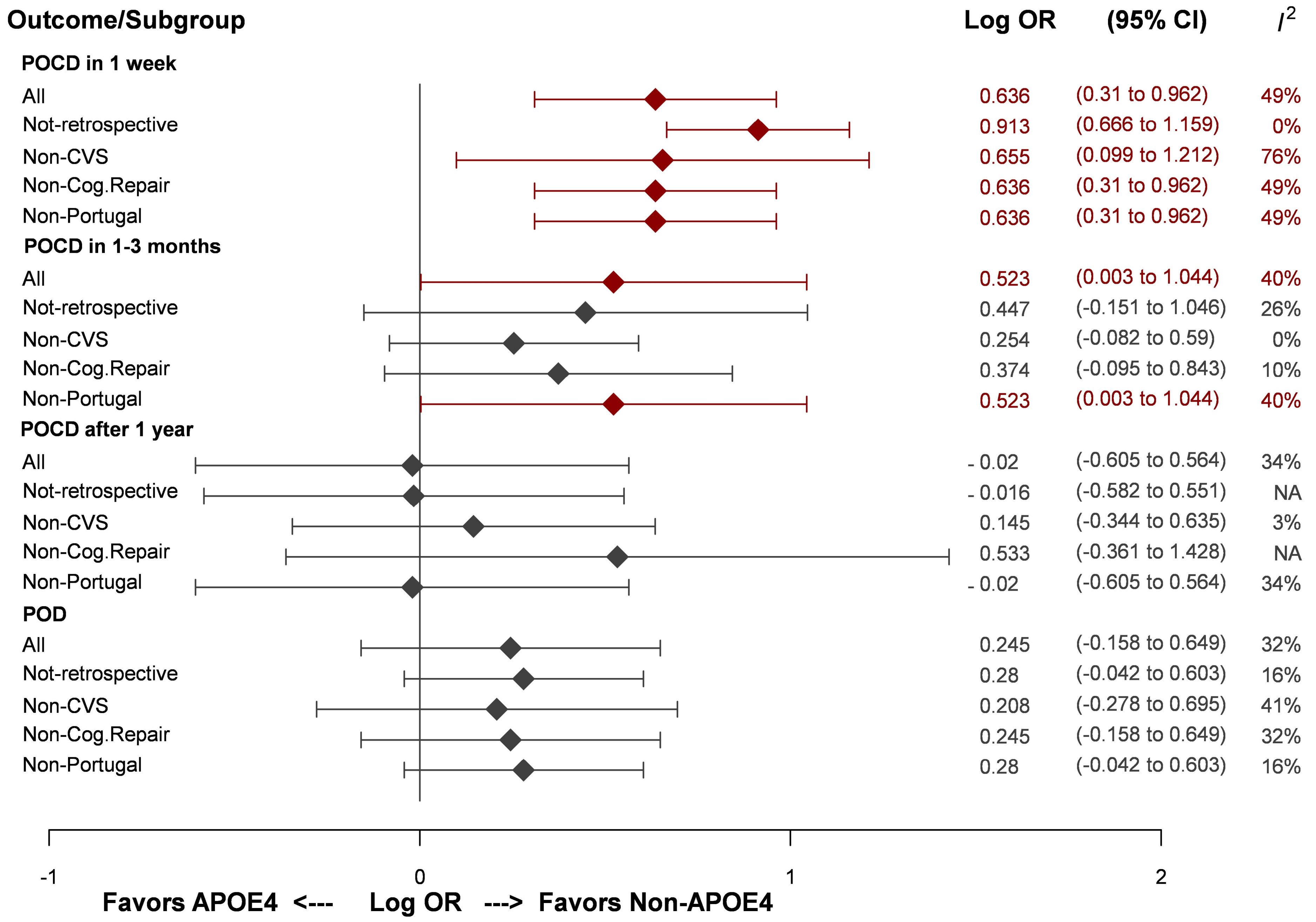

Supplement: S1 Fig — (PDF) [file pone.0282214.s004.pdf]
