# Supplementary figures and images for: Apolipoprotein E4 allele is genetically associated with risk of the short- and medium-term postoperative cognitive dysfunction: A meta-analysis and trial sequential analysis
Source: PLoS One. 2023 Feb 24;18(2):e0282214. doi: 10.1371/journal.pone.0282214 (PMC9955600; doi:10.1371/journal.pone.0282214)

**S4 Fig. Trial sequential analysis of the incidence of POCD or POD.**

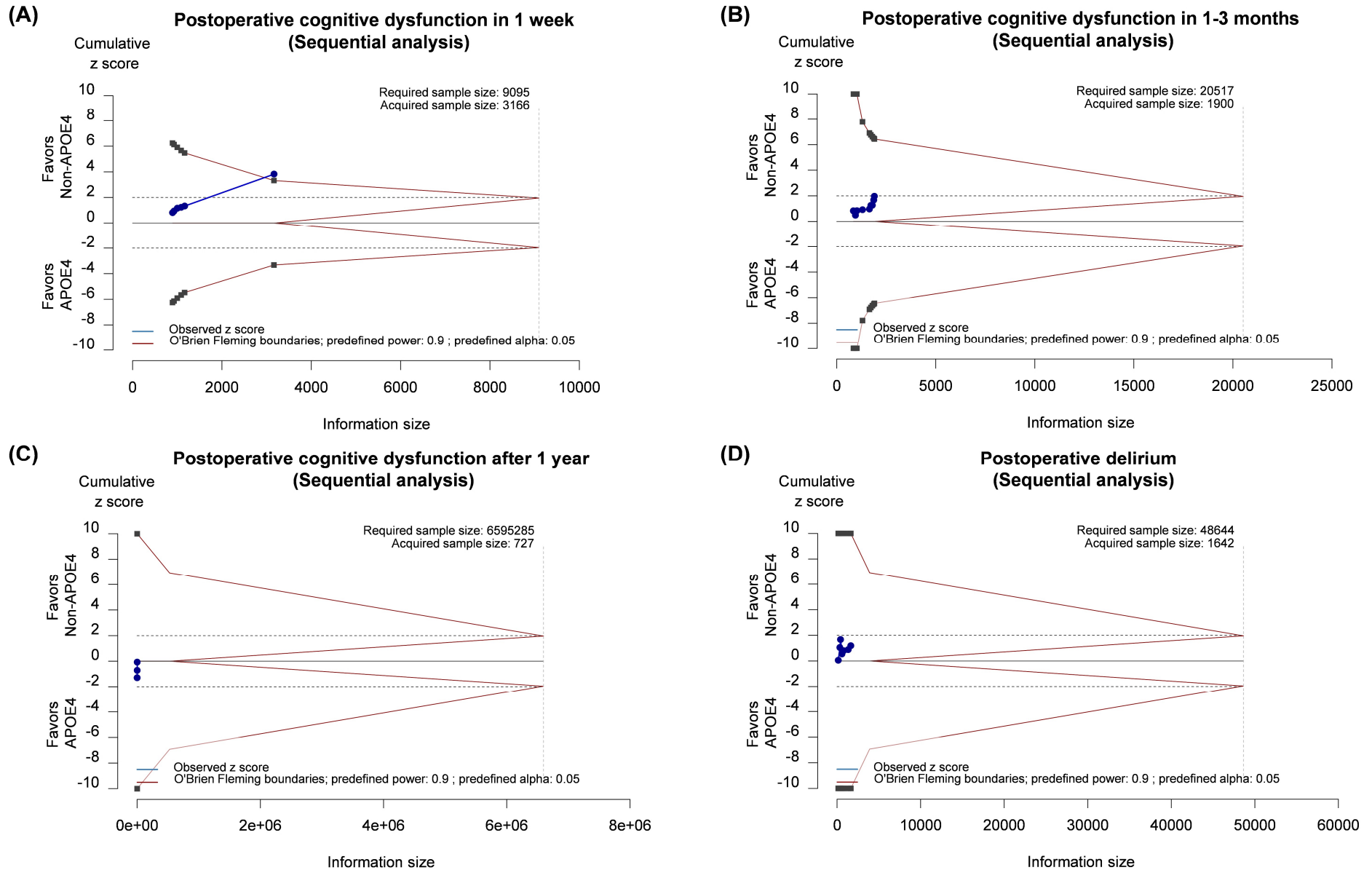

Supplement: S2 Fig — (PDF) [file pone.0282214.s005.pdf]
